# The neuropeptide PACAP alleviates *T. gondii* infection-induced neuroinflammation and neuronal impairment

**DOI:** 10.1186/s12974-022-02639-z

**Published:** 2022-11-19

**Authors:** Caio Andreeta Figueiredo, Henning Peter Düsedau, Johannes Steffen, Stefanie Ehrentraut, Miklos P. Dunay, Gabor Toth, Dora Reglödi, Markus M. Heimesaat, Ildiko Rita Dunay

**Affiliations:** 1 Institute of Inflammation and Neurodegeneration, Health Campus Immunology, Infectiology and Inflammation (GC-I3), Otto-Von-Guericke University, Magdeburg, Germany; 2 Department and Clinic of Surgery and Ophthalmology, University of Veterinary Medicine, Budapest, Hungary; 3 Department of Medical Chemistry, University of Szeged, Budapest, Hungary; 4 Department of Anatomy, MTA-PTE PACAP Research Team and Szentagothai Research Center, University of Pecs Medical School, Pecs, Hungary; 5 Institute of Microbiology, Infectious Diseases and Immunology, Charité - University Medicine Berlin, Berlin, Germany; 6 Center for Behavioral Brain Sciences – CBBS, Magdeburg, Germany

**Keywords:** PACAP, Neuroinflammation, *Toxoplasma gondii*, Cerebral toxoplasmosis, Immunomodulation, Neuroprotection

## Abstract

**Background:**

Cerebral infection with the protozoan *Toxoplasma gondii* (*T. gondii*) is responsible for inflammation of the central nervous system (CNS) contributing to subtle neuronal alterations. Albeit essential for brain parasite control, continuous microglia activation and recruitment of peripheral immune cells entail distinct neuronal impairment upon infection-induced neuroinflammation. PACAP is an endogenous neuropeptide known to inhibit inflammation and promote neuronal survival. Since PACAP is actively transported into the CNS, we aimed to assess the impact of PACAP on the *T. gondii*-induced neuroinflammation and subsequent effects on neuronal homeostasis.

**Methods:**

Exogenous PACAP was administered intraperitoneally in the chronic stage of *T. gondii* infection, and brains were isolated for histopathological analysis and determination of pathogen levels. Immune cells from the brain, blood, and spleen were analyzed by flow cytometry, and the further production of inflammatory mediators was investigated by intracellular protein staining as well as expression levels by RT-*q*PCR. Neuronal and synaptic alterations were assessed on the transcriptional and protein level, focusing on neurotrophins, neurotrophin-receptors and signature synaptic markers.

**Results:**

Here, we reveal that PACAP administration reduced the inflammatory foci and the number of apoptotic cells in the brain parenchyma and restrained the activation of microglia and recruitment of monocytes. The neuropeptide reduced the expression of inflammatory mediators such as IFN-γ, IL-6, iNOS, and IL-1β. Moreover, PACAP diminished IFN-γ production by recruited CD4^+^ T cells in the CNS. Importantly, PACAP promoted neuronal health via increased expression of the neurotrophin BDNF and reduction of p75^NTR^, a receptor related to neuronal cell death. In addition, PACAP administration was associated with increased expression of transporters involved in glutamatergic and GABAergic signaling that are particularly affected during cerebral toxoplasmosis.

**Conclusions:**

Together, our findings unravel the beneficial effects of exogenous PACAP treatment upon infection-induced neuroinflammation, highlighting the potential implication of neuropeptides to promote neuronal survival and minimize synaptic prejudice.

**Supplementary Information:**

The online version contains supplementary material available at 10.1186/s12974-022-02639-z.

## Background

Neurological disorders are mainly associated with the development of neuroinflammation in response to autoimmune recognition, neurodegenerative accumulation of toxic metabolites, or due to infectious diseases [1, 2]. Most frequent infections of the central nervous system (CNS) are caused by viral, bacterial and fungal pathogens, although neurotropic protozoan parasites such as *Toxoplasma gondii* (*T. gondii*) can cause cerebral toxoplasmosis, a persistent subclinical to severe neuroinflammatory disease [3, 4]. It is estimated that one-third of the world’s human population is infected with *T. gondii* and the seroprevalence is even higher in other mammals [5–7]. Once invading neuronal tissue, the parasites evoke an efficient pro-inflammatory immune response with release of tumor necrosis factor (TNF), interleukin (IL)-6, IL-1β, and nitric oxide (NO) by activated resident microglia and astrocytes [8, 9]. The local immune response is intensified by the recruitment of peripheral lymphoid and myeloid cells to the brain, which are crucial for parasite control mainly via interferon-gamma (IFN-γ) production [10–13]. The persistent infection in the brain establishes an ongoing neuroinflammation for the host lifetime, and is able to modify neuronal function and synapse composition [9, 14–16]. Indeed, neurological and behavior alterations upon *T. gondii* infection have been highlighted in rodent and human studies [17–20], and associated to altered cognitive function [21] and several neuropsychiatric conditions including depression, suicidal behavior, and schizophrenia [22–24]. As no current therapy is able to completely eliminate the parasites from the CNS, new therapeutic strategies are necessary to reduce infection-induced neuroinflammation, preventing neuronal death and minimizing synaptic prejudice [25].

The neuropeptide PACAP (Pituitary Adenylate Cyclase-Activating Polypeptide) has been described to strongly modify inflammation and clinical symptomatology in different rodent models of inflammatory diseases [26, 27]. The neuropeptide also acts as a neurotransmitter, neuromodulator, and neurotrophic factor [28, 29]. The full-length biologically active form of PACAP (PACAP1-38) is a 38-amino-acid neuropeptide commonly described as being widely expressed in the CNS of mammals [28, 30]. According to recent annotations in the Human Protein Atlas program database, single-cell RNA analyses have shown expression of PACAP (codified by *Adcyap1* gene) mostly by granulocytes and excitatory neurons, and in lesser manner (10 fold) by specific epithelial cells and fibroblasts, T cells, microglia, and plasma cells [31, 32]. PACAP employs its biological activity through binding to three widely distributed G protein-coupled receptors (GPCRs) named PAC1R, VPAC1R and VPAC2R [33], which triggers a series of intracellular signaling pathways, mainly involving production of cyclic adenosine monophosphate (cAMP) [34, 35]. PACAP receptors have different affinities for PACAP, with PAC1R possessing the highest affinity [36]. In addition, the receptors are differently expressed among immune cells, for example PAC1R and VPAC1R are constitutively expressed by monocytes, peritoneal macrophages and microglia, while T cells do not express PAC1R. VPAC2R is rarely detected under steady-state, but its expression is induced upon inflammation [37–39]. Despite neuroprotective potential in several neurological disorders such as Parkinson’s [40, 41], Alzheimer’s [42], and Huntington’s disease [43], as well as traumatic brain injury [44], clinical studies targeting PACAP signaling have been conducted addressing nociception upon nephrotic syndrome, cluster headache, migraine, and major depression [45, 46]. In fact, immuno-modulatory properties of PACAP were found to be ambiguous, transiting from inhibition to stimulation depending on the experimental conditions and cell types [47], and therefore they request further investigation.

Our previous studies applying the acute infection-induced inflammation model have demonstrated that the resolution of intestinal inflammation and enhanced phagocytic capacity of mononuclear cells was achieved by exogenous administration of PACAP [39, 48, 49]. As PACAP has shown beneficial effects in neurological and inflammatory diseases and given its active transport into the brain across the blood–brain barrier (BBB) [50], we set out to investigate the effects of exogenous PACAP administration during cerebral toxoplasmosis, a *T. gondii*-induced neuroinflammation model. Our results point toward amelioration of brain inflammation, with reduced recruitment of myeloid cells and marked reduction of microglia activation and cytokine production, without triggering uncontrolled parasite burden. In addition, our results provide evidence that PACAP promotes neuronal health via BDNF/p75^NTR^ axis modulation, favoring a less dysfunctional neuronal network of glutamatergic and GABAergic signaling, which is specifically affected during *T. gondii*-induced neuroinflammation.

## Methods

### Mice and *T. gondii* infection-induced neuroinflammation model

Experiments were conducted with female C57BL/6J mice (8–14 weeks old, purchased from Janvier, Cedex, France). All animals were group-housed in a 12-h day/night cycle at 22 °C with free access to food and water under specific pathogen-free conditions and according to institutional guidelines approved by the Animal Studies Committee of Saxony-Anhalt. In order to investigate *T. gondii*-induced neuroinflammation, mice were infected by intraperitoneal (i.p.) injection of cysts of the type II ME49 strain, previously harvested from brains of female NMRI mice infected i.p. with *T.* *gondii* cysts 6–12 months before, as described elsewhere [51]. Briefly, brains isolated from NMRI mice were mechanically homogenized in 1 ml sterile phosphate-buffered saline (PBS), and the number of cysts was determined in ≥ 80 µl homogenate using a bright field microscope. The homogenate was diluted in sterile PBS, and animals were infected i.p with two cysts. In our model, cerebral toxoplasmosis is established after 3 weeks post-infection, as described elsewhere [11, 15, 16, 52].

### PACAP treatment and organ collection

For all experiments, PACAP1-38 (the full-length 38-amino acid peptide, simply referred here as PACAP) was synthesized using solid phase strategy combined with the *Fmoc* chemistry methodology at the Department of Medical Chemistry, University of Szeged (Hungary) as previously described [53]. To investigate the effect during *T. gondii*-induced neuroinflammation, 100 µg of PACAP in phosphate-buffered saline (PBS; 5 mg per kg body weight, 22 nM per mouse) or PBS (for non-treated controls) were administered i.p. on alternating days for one week, from the third to fourth week post-infection (4 administrations in total). One day after the last treatment, animals were killed, blood samples obtained by puncture of the vena cava inferior and diluted in ice-cold PBS for flow cytometric analysis. Next, animals were transcardially perfused with 60 ml sterile ice-cold PBS, and brain, lung, and spleen were collected and stored in sterile ice-cold PBS for flow cytometric analysis, or stored in RNAlater® (Sigma, Germany) for nucleic acid isolation and subsequent *q*PCR or RT-*q*PCR analysis. Samples stored in RNAlater® were kept at 4 °C overnight and then stored at −80 °C.

### Cell isolation

Isolation of immune cells from brain was performed as previously described [52, 54]. Briefly, brain hemispheres were mechanically homogenized in dissection buffer (HBSS, Gibco™, Germany), supplemented with 50 mM glucose (Roth, Germany) and 13 mM HEPES (pH 7.3, Sigma), using a glass potter, and then filtered through a 70-μm cell strainer (Falcon®, Corning, Germany). The cell suspension was washed (400 g, 10 min, 4 °C) with PBS and fractionated on a discontinuous 30%–70% isotonic Percoll® gradient (GE Healthcare, Germany) (800 g, 30 min without brake, 4 °C). After the removal of myelin debris, cells in the interphase comprising mononuclear cells were isolated and washed with FACS buffer (PBS w/o Ca^2+^/Mg^2+^, 2% v/v fetal bovine serum (FBS), 10 mM HEPES, 0.1% sodium azide), centrifuged (400 g, 10 min, 4 °C) and immediately used for flow cytometric analysis. To isolate immune cells from spleens, organs were homogenized and sieved through a 40-μm cell strainer (Falcon®, Corning). Isolated cells from spleen and blood samples were treated with erythrocyte lysis buffer (eBioscience, Germany), and washed twice with FACS buffer (400 g, 10 min, 4 °C) before staining and flow cytometric analysis.

### Flow cytometric analysis

For surface staining, single-cell suspensions were first incubated with ZOMBIE NIR™ fixable dye (BioLegend, San Diego, CA) for live/dead discrimination. To prevent unspecific binding of antibodies, anti-mouse CD16/32 antibody (clone 93, BioLegend) was applied to cells before staining with fluorochrome-conjugated antibodies against cell surface markers in FACS buffer. CD11b (clone M1/70), Ly6C (clone HK1.4), CD4 (clone GK1.5) and CD8a (clone 53–6.7) were all purchased from eBioscience. CD45 (30-F11), Ly6G (1A8), CD3 (17A2), MHCII I-A/I-E (clone M5/114.15.2) were purchased from BioLegend. Cells were incubated for 30 min at 4 °C, washed (400 g, 10 min, 4 °C) and subsequently analyzed. Fluorescence Minus One (FMO) controls were used to determine the positive signals for each conjugated antibody.

Flow cytometric analysis of intracellular cytokines was performed as described elsewhere [52]. Briefly, isolated immune cells from the brain were re-stimulated with Toxoplasma Lysate Antigen (TLA) at 20 µg/ml for 2 h [55]. Then, Brefeldin A (10 µg/ml, GolgiPlug, BD Biosciences) and Monensin (10 µg/ml GolgiStop, BD Biosciences) were added and the cells were further incubated for additional 4 h. After one washing step with FACS buffer (400 g, 10 min, 4 °C), cells were incubated with ZOMBIE NIR™ and anti-mouse CD16/32 antibody prior to surface staining described above. Afterwards, cells were fixed in 4% paraformaldehyde (PFA) for 15 min and subsequently permeabilized with Permeabilization Buffer (BioLegend). Intracellular proteins were stained with fluorochrome-conjugated antibodies against IFN-γ (XMG1.2; eBiosciences), iNOS (clone 6, BD Biosciences), IL-1β (clone NJTEN, eBiosciences), and TNF (clone MP6-XT22, BioLegend) in Permeabilization Buffer. Matching isotype controls were used to assess the level of unspecific binding. Data were acquired using the Attune™ NxT flow cytometer (Thermo Fisher; Germany) and analyzed using FlowJo™ (v10, LLC, BD Life Sciences, USA). A minimum of 2 × 10^5^ cells per sample was acquired and analyzed.

### DNA and RNA isolation

For DNA and RNA isolation, brains, lungs, and spleens were removed from RNAlater® and homogenized in TriFast™ (Peqlab, VWR, LLC, Germany) or lysis buffer using BashingBeads Lysis tubes (Zymo Research Europe, Germany) and BeadBug 6 homogenizer (Biozym, Germany). DNA and RNA were isolated from the homogenate by isopropanol precipitation using Total RNA Kit peqGOLD® (Peqlab, VWR) or isolated using AllPrep DNA/RNA Mini Kit (Qiagen, Germany) according to the manufacturer’s instructions. Concentration and purity of DNA and RNA was determined using NanoDrop 2000 spectrophotometer (Thermo Fisher) and samples stored at −80 °C until further use.

### *q*PCR

Parasite burden was assessed in triplicates using 30 ng of isolated DNA, FastStart Essential DNA Green Master (Roche, Germany) and LightCycler® 96 System (Roche) as described elsewhere [39]. Thermal-cycling parameters were set as follows: initial activation (95 °C, 10 min), 45 amplification cycles consisting of denaturation (95 °C, 15 s), annealing (60 °C, 15 s), and elongation (72 °C, 15 s). The DNA target was the published sequence of the highly conserved 35-fold-repetitive B1 gene of *T. gondii* (*TgB1*) [56, 57], and murine argininosuccinate lyase (*Asl*) was used for normalization [58]. Primers listed in Table1 were synthetized by Tib MolBiol (Germany) and used at 300 nM final concentration.

**Table 1:** Oligonucleotide primers used for qPCR and RT-qPCR based on SYBR Green

Protein	Gene		Sequence (5′–3′)
ASL	*Asl*	Fw	TCTTCGTTAGCTGGCAACTCACCT
		Rv	ATGACCCAGCAGCTAAGCAGATCA
BAG1*	*Bag1*	Fw	GACGTGGAGTTCGACAGCAAA
		Rv	ATGGCTCCGTTGTCGACTTCT
GAPDH	*Gapdh*	Fw	TTGTCAAGCTCATTTCCTGGTATG
		Rv	TGGTCCAGGGTTTCTTACTCCTT
SAG1*	*Sag1*	Fw	ATCGCCTGAGAAGCATCACTG
		Rv	CGAAAATGGAAACGTGACTGG
B1*	*B1*	Fw	GCATTGCCCGTCCAAACT
		Rv	AGACTGTACGGAATGGAGACGAA
GAD65	*Gad2*	Fw	GGAATCTTTTCTCCTGGTGGC
		Rv	CACTCACCAGGAAAGGAACAAA
GAD67	*Gad1*	Fw	CTGAACCGAGCCTGTTCCTG
		Rv	TCATACGTTGTAGGGCGCAG
GAT-1	*Slc6a1*	Fw	GTTGGACTGGAAAGGTGGTCT
		Rv	AGCTTTCGGAAGTTGGGTGT
GAT-2	*Slc6a13*	Fw	GCCTCGGGAACAACCAGTAAT
		Rv	GACAGGGATGCCACAGGTAAA
GAT-3	*Slc6a11*	Fw	CGGCTGGGTATATGGAAGCA
		Rv	GCCCCAAGCAGGATATGTGT
VGAT	*Slc32a1*	Fw	CACTGCGACGATCTCGACTT
		Rv	CACGAACATGCCCTGAATGG
PAC1R	*Adcyap1r1*	Fw	GGCTGTGCTGAGGCTCTACTTTG
		Rv	AGGATGATGATGATGCCGATGA
VPAC1R	*Vipr1*	Fw	GATGTGGGACAACCTCACCTG
		Rv	TAGCCGTGAATGGGGGAAAAC
VPAC2R	*Vipr2*	Fw	GCGGTGTCTGGGACAACATC
		Rv	CTGTGACATTTTCCCCAACGT

*T. gondii

### RT-*q*PCR

Transcription levels of inflammatory mediators, host-defense factors, neurotrophins, neurotrophin receptors, and synaptic proteins were assessed in triplicates using 30 ng isolated RNA, TaqMan® RNA-to-CT™ 1-Step Kit (Applied Biosystems, Germany), and LightCycler® 96 (Roche, Germany) as described elsewhere [39]. TaqMan® Gene Expression Assays (Thermo Fisher) utilized are listed in Table 2. *Hprt* was chosen as reference gene and relative mRNA levels were determined by the ratio *gene of interest/reference gene* and subsequently normalized to mean values of control group. To determine parasite life-stage conversion, the expression of stage-specific genes for tachyzoites (*Sag1*) and bradyzoites (*Bag1*) [59] was evaluated using Power SYBR® Green RNA-to-CT™ 1-Step Kit (Thermo Fisher). Samples were analyzed in triplicates (50 ng of isolated mRNA per reaction) using LightCycler® 96 (Roche) and the following parameters: reverse transcription (48 °C, 30 min), inactivation (95 °C, 10 min) followed by 55 cycles of denaturation (95 °C, 15 s), annealing/extension (60 °C, 1 min), and melting curve analysis. The primers used for *Sag1* (SAG1), *Bag1* (BAG1) and murine *Gapdh* are listed in Table 1, and were synthetized by Tib MolBiol and used at 100 nM final concentration. Expression of *Gapdh* was chosen as reference gene and relative mRNA levels were determined by the ratio *gene of interest/reference gene* and subsequently normalized to mean values of control group. Similarly, transcriptional levels of glutamate decarboxylase enzymes, GABA transporters, and PACAP receptors were determined as described above, using Power SYBR® Green RNA-to-CT™ 1-Step Kit (Thermo Fisher) with 30 ng mRNA per reaction and primers listed in Table 1 used at 200 nM final concentration.

**Table 2:** Oligonucleotide primers used for RT-qPCR based on TaqMan®

Protein	Gene	Gene expression assay
BDNF	*Bdnf*	Mm04230607_s1
GABAα1	*Gabra1*	Mm00439046_m1
GBP-1	*Gbp2b*	Mm00657086_m1
HPRT	*Hprt*	Mm01545399_m1
IFN-γ	*Ifng*	Mm00801778_m1
Irgm3	*Igtp*	Mm00497611_m1
IL-1β	*Il1b*	Mm00434228_m1
IL-6	*Il6*	Mm00446190_m1
Irgm1	*Irgm1*	Mm00492596_m1
NGF	*Ngf*	Mm00443039_m1
P75^NTR^	*Ngfr*	Mm01309638_m1
iNOS	*Nos2*	Mm00440485_m1
Nt-3	*Ntf3*	Mm01182924_m1
TrkA	*Ntrk1*	Mm01219406_m1
TrkB	*Ntrk2*	Mm00435422_m1
VGLUT1	*Slc17a7*	Mm00812886_m1
EAAT2	*Slc1a2*	Mm01275814_m1
TNF	*Tnf*	Mm00443258_m1

### Histopathology and immunohistochemistry

For histopathological analysis, brains were fixed with 4% paraformaldehyde (PFA) at 4 °C immediately after removal, and embedded in paraffin. Sagittal sections were prepared at 5 µm. To evaluate the general pathological changes and the number of inflammatory foci sections were stained using hematoxylin and eosin (H&E). Brain sections were further analyzed by in situ immunohistochemistry for quantification of F4/80^+^ macrophages and apoptotic cells. Primary antibodies against F4/80 (1:50, clone BM8, eBioscience) and cleaved Caspase-3 (1:200, Cell Signaling, USA) were used. A minimum of 2–4 sagittal sections were analyzed per animal. Axiovert 200 bright field microscope equipped with an AxioCam ERc 3 digital camera and ZEN software (ZEISS, Germany) were used. The number of positively stained cells was assessed in 10 randomly chosen images (40× objective) within brain cortical regions. The total number of inflammatory foci was assessed using a 10× objective across the brain sections. Slides were analyzed in an independent and blinded manner.

### Western blot analysis

Proteins were isolated from whole brain homogenates previously prepared with TriFast™ (Peqlab, VWR, LLC, Germany) using an optimized method described elsewhere [60]. Protein concentrations were determined using Bradford reagent (Bio-Rad Protein Assay, Bio-Rad, Germany) and read at *λ* = 595 nm using SpectraMax M5e (Molecular Devices LLC). Total of 15 µg of protein from each sample were separated by SDS-PAGE 10%, and transferred to nitrocellulose membrane (Amersham™ Protran™, GE Healthcare, Germany). Membranes were blocked for 2 h in ROTI®Block solution diluted in Tris-Buffered Saline (TBS) containing 0.1% (v/v) Tween-20 (Roth, Germany) (TBS-T), and subsequently incubated overnight at 4 °C with primary antibodies in the same blocking solution. Accordingly, GAD65 (Synaptic System, SYSY, Germany, #198,104, 1:1000), GAD67 (SYSY, #198,211, 1:500), TrkB (CST, #4603, 1:1000), beta-III-Tubulin (Sigma, #T8660, 1:1000) and beta-actin (CST, #13E5, 1:1000) were used. In the following day, membranes were washed three times (10 min each) in TBS-T and then incubated for 1 h room temperature with secondary HRP-conjugated antibodies diluted in TBS-T at 1:10,000. Anti-guinea pig (Invitrogen, #A18769), anti-rabbit (Jackson ImmunoResearch, #111–035-144) and anti-mouse (Jackson ImmunoResearch, #115–035-068) were used. Membranes were revealed using SuperSignal™ West kit (Thermo Fisher, #34,078 and #34,095) and densitometric analysis of the blots were performed using ImageJ/Fiji.

### Statistical analysis

Results were statistically analyzed using GraphPad Prism 7 (GraphPad Software Inc., USA). Data were considered statistically significant with *p* ≤ 0.05. All data are presented as arithmetic mean with standard error of the mean (SEM), collected from at least two independent experiments. For histopathological data, *q*PCR and RT-*q*PCR, data were analyzed by non-parametric Mann–Whitney *U*-test. For flow cytometric and western blot analyses, data were analyzed by two-tailed unpaired *t*-test.

## Results

### PACAP alleviates brain pathology upon cerebral *T. gondii* infection

**Fig. 1:**
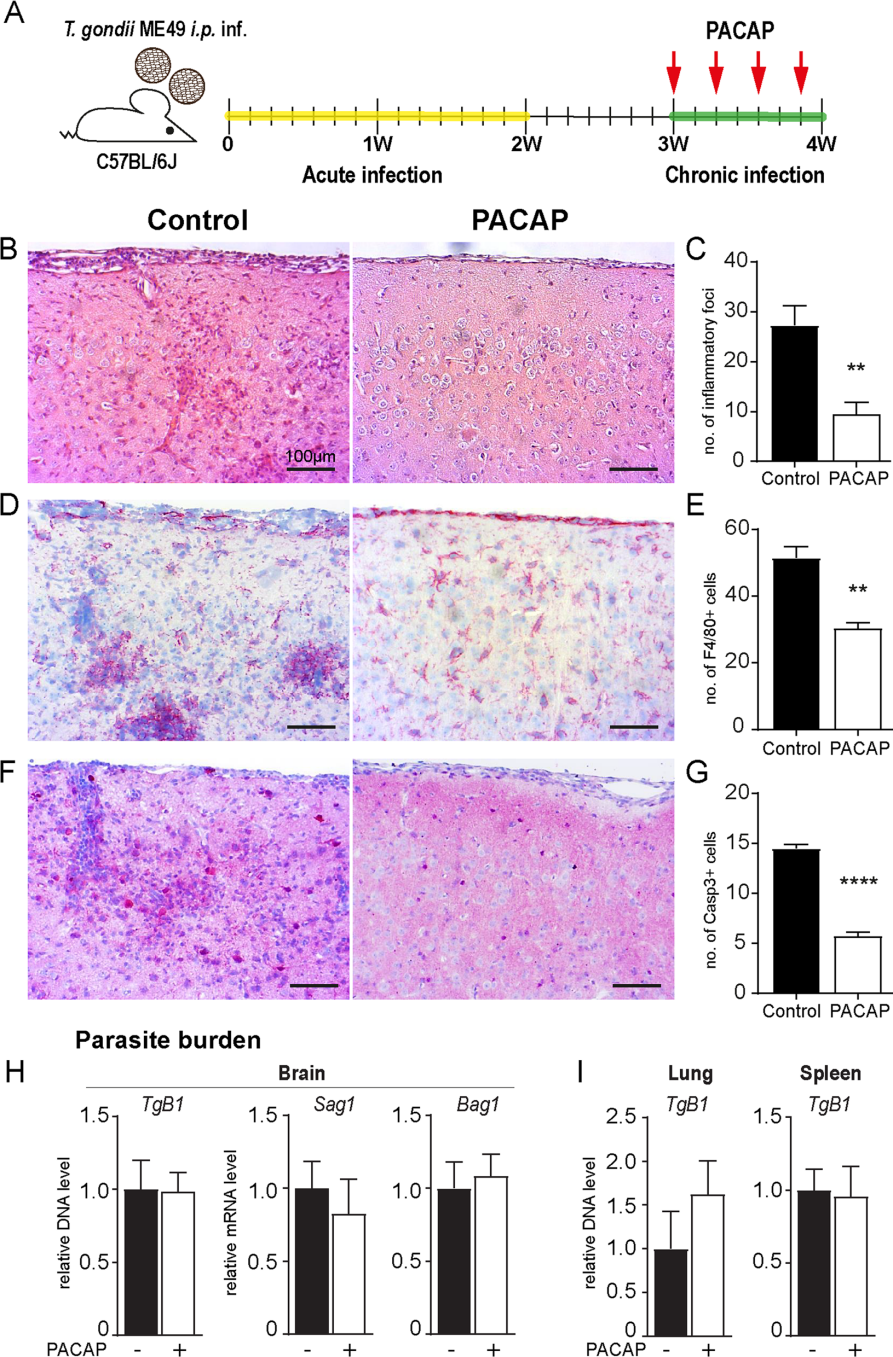
PACAP administration alleviates severe brain pathology upon *T. gondii* encephalitis. **A** Experimental design and PACAP administration schedule. Mice were infected with 2 cysts of *T. gondii* (type II strain ME49) and treated with 100 µg PACAP i.p. on alternating days starting at 3 weeks post-infection; yellow indicates acute infection, followed by an intermediated phase (2 to 3 weeks post-infection, not indicated), and green indicates chronic infection. **B**, **D**, **F** Histological brain sections from infected control animals (left column) and PACAP-treated animals (right column). **B** H&E staining and **C** bar charts show number of inflammatory foci quantified across 10 consecutive brain sections per mouse. **D** F4/80 staining and **E** bar charts show the mean of F4/80^+^ cells across 10 random cortical areas per mouse. **F** Caspase-3 (Casp3) staining and **G** bar charts show the mean of Casp3^+^ cells across 10 random cortical areas per mouse. Data show mean + SEM, n = 4, **p < 0.01, ***p < 0.001, ****p < 0.0001 (unpaired two-tailed t-test). Scale bars = 100 µm. (H) Parasite burden was determined by *q*PCR analysis of TgB1 abundance, and RT-*q*PCR of *Sag1* and *Bag1* gene expression in brain homogenate. (I) Parasite burden in peripheral organs determined by TgB1 abundance. Bar charts present results normalized to mean values of control group as mean + SEM obtained in two independent experiments and analyzed together, *n* = 4–5 (Mann–Whitney *U*-test); Control (black bars) and PACAP-treated (white bars)

*T. gondii* neuroinfection is characterized by activation of glial cells and recruitment of immune cells to the CNS. Albeit promotion of pathogen clearance, the inflammatory responses also result in severe brain lesions and destruction of neuronal tissue [61]. To assess the effects of exogenous PACAP administration upon cerebral toxoplasmosis, we utilized a murine model susceptible to chronic progressive infection [9]. Therefore, C57BL/6 J animals were infected with two cysts of type II ME49 parasite strain. After the initial acute infection, 100 µg of PACAP or vehicle only (for non-treated controls) were administered i.p. starting three weeks post-infection (Fig. 1A). One day after the last treatment, brain tissue was collected for histopathological analyses (Fig. 1B–G). In non-treated control- and PACAP-treated animals, we detected parenchymal hemorrhage and inflammatory foci of cellular infiltrates. In contrast, the number of inflammatory foci was strongly reduced in PACAP-treated mice (Fig. 1B–C). Additionally, PACAP treatment resulted in reduced numbers of F4/80^+^ cells (Fig. 1D, E), which was paralleled by lower number of brain apoptotic cells (Casp3^+^) (Fig. 1F, G). Hence, these results point towards an anti-inflammatory and neuroprotective effect of PACAP. In order to assess whether this amelioration of inflammation negatively affected parasite control, we determined *T. gondii* burden in the brain of mice. Our results revealed an unaltered parasite burden, and no alteration of parasite life-stage conversion upon PACAP treatment (Fig. 1H). Similarly, parasite burden in peripheral organs was also not affected (Fig. 1I). Thus, PACAP treatment of chronically infected mice suggested amelioration of neuroinflammation without hampering parasite control. Accordingly, we hypothesize that PACAP effects are likely due to modulation of immune cell recruitment to the CNS and their activation status.

### PACAP restricted myeloid cell recruitment and reduced microglia and monocyte activation

**Fig. 2:**
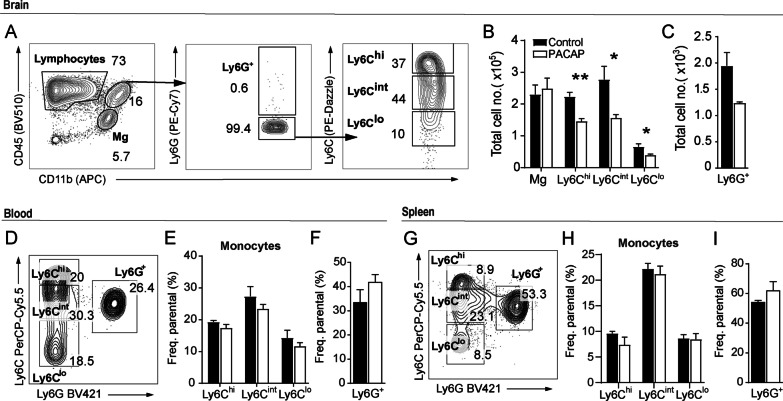
Analysis of microglia and myeloid immune cell subsets upon PACAP treatment. Immune cells were isolated from brain, blood and spleen, and analyzed by flow cytometry. Only single, live immune cells were considered for analysis. **A** Representative gating strategy applied for discrimination of CD45^hi^CD11b^−^lymphocytes and CD45^int^CD11b^int^ microglia (Mg). CD45^hi^CD11b^hi^ myeloid cells (upper right gate) were further discriminated into Ly6G^+^ neutrophils, and Ly6G^−^ mononuclear cells were differentiated according to Ly6C expression in Ly6C^hi^ inflammatory monocytes, Ly6C^int^ monocyte-derived DCs and Ly6C^lo^ monocyte-derived macrophages. **B**, **C** Bar charts show total cell numbers of microglia and myeloid immune cell subsets in the brain. **D**, **G** Myeloid cells from blood and spleen were first identified as CD11b^+^ (not shown). Contour plots show the classification of selected cells into monocyte subsets (according to Ly6C expression) and Ly6G^+^ neutrophils. Bar charts compare the cell frequency in the blood (**E**, **F**) and spleen samples (**H**, **I**) as percentages from parent population. Data show individual values as mean + SEM, *n* = 4–5, *p < 0.05, **p < 0.01 (unpaired two-tailed t-test) from a representative experiment; Control (black bars) and PACAP-treated (white bars)

**Fig. 3:**
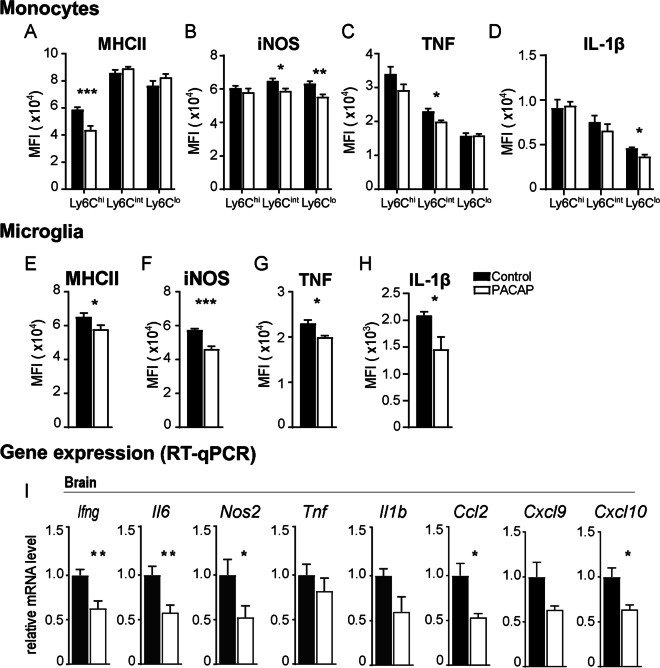
Monocyte and microglia activation and inflammatory mediators. Previously identified brain monocytes subsets and microglia were analyzed by flow cytometry for surface expression of MHCII (**A**, **E**), and for intracellular production of iNOS, TNF and IL-1β (**B**–**D**, **F**–**H**). Bar charts show values of median fluorescence intensity (MFI) previously defined for each population and marker, based on their respective fluorescence-minus-one (FMO) controls (not shown). Data represent mean values + SEM, *n* = 4–5, *p < 0.05, **p < 0.01, ***p < 0.001 (unpaired two-tailed *t*-test); **I** gene expression of inflammatory mediators and chemokines was assessed in whole brain homogenate. Bar charts represent mean values + SEM obtained in two independent experiments and analyzed together, *n* = 4–5 per experiment *p < 0.05, **p < 0.01 (Mann–Whitney *U*-test); Control (black bars) and PACAP-treated (white bars)

In previous studies, we demonstrated that neutrophils and myeloid cells continuously infiltrate the CNS during cerebral toxoplasmosis [11, 12]. As suggested by the histopathological analysis, we hypothesize that PACAP ameliorates neuroinflammation and reduces cellular death by restraining immune cell infiltration to the brain. For further investigation, we isolated immune cells from the brain of *T. gondii*-infected animals, and assessed the differences between PACAP-treated animals and infected non-treated controls regarding immune cell recruitment and activation. CD45 and CD11b were used as markers to identify lymphocytes (CD45^+^ CD11b^low^, upper left gate), myeloid cells (CD45^+^ CD11b^high^, upper right gate) and activated microglia (CD45^int^ CD11b^int^, lower right gate) (Fig. 2A). Myeloid cells were further discriminated by Ly6G expression, providing differentiation between neutrophil granulocytes (Ly6G^+^ CD11b^+^, upper gate) and mononuclear cells (Ly6G^−^ CD11b^+^, lower gate). Then, mononuclear cells were classified according to Ly6C expression, and subdivided in CD11b^+^ Ly6C^hi^ inflammatory monocytes (upper gate), CD11b^+^ Ly6C^int^ monocytes-derived dendritic cells (DCs) (middle gate) and CD11b^+^ Ly6C^lo^ monocyte-derived macrophages (lower gate) as previously described by our group [11]. When compared to control, PACAP-treated mice showed a reduced number of recruited myeloid cell subsets (Fig. 2B). Also, the recruitment of Ly6G^+^ neutrophil granulocytes was reduced without reaching significance (*p* = 0.065) in PACAP group (Fig. 2C). Next, we hypothesized that the reduction of cell recruitment to the brain of PACAP-treated mice might simply be a reflection of diminished peripheral immune response. Thus, we investigated the immune cell compartment in blood (Fig. 2D-–F) and spleen (Fig. 2G–I), and no changes in myeloid cell frequency were detected between control and PACAP group. Further on, we assessed whether PACAP would modulate the activation status of monocytes subsets in terms of MHCII expression, which is crucial for a potent inflammatory adaptive immune response [62]. We detected reduced MHCII expression by Ly6C^hi^ inflammatory monocytes in the brain (Fig. 3A). In addition, we assessed the production of NO, a key role pro-inflammatory mediator measured via expression of inducible NO synthase (iNOS). Levels of iNOS were reduced on Ly6C^int^ and Ly6C^lo^ monocyte subsets upon PACAP treatment (Fig. 3B). Moreover, the production of pro-inflammatory cytokines TNF and IL-1β was reduced in Ly6C^int^ and Ly6C^lo^ subsets, respectively (Fig. 3C, D). Next to the altered activation of infiltrating myeloid cells, we found that the activation status of resident microglia was changed upon PACAP, indicated by reduced expression of MHCII, and intracellular production of iNOS, TNF, and IL1-β (Fig. 3E–H). At last, we assessed the overall impact of PACAP in the transcriptional level of inflammatory mediators and chemokines in the entire brain tissue, and we detected reduced expression of IFN-γ, IL-6, iNOS (*Nos2*), IL-1β, CCL2, CXCL9 and CXCL10 (Fig. 3I). Regarding modulation of PACAP receptors levels in overall brain, all receptors presented higher expression upon infection, and only PAC1R was reduced upon PACAP treatment (Additional file 1A). In sum, PACAP impacted the recruitment of myeloid cells to brain, reduced the expression of inflammatory mediators and chemokines in the brain parenchyma.

### PACAP diminished IFN-γ production by recruited CD4^+^ T cells in infected brains

**Fig. 4:**
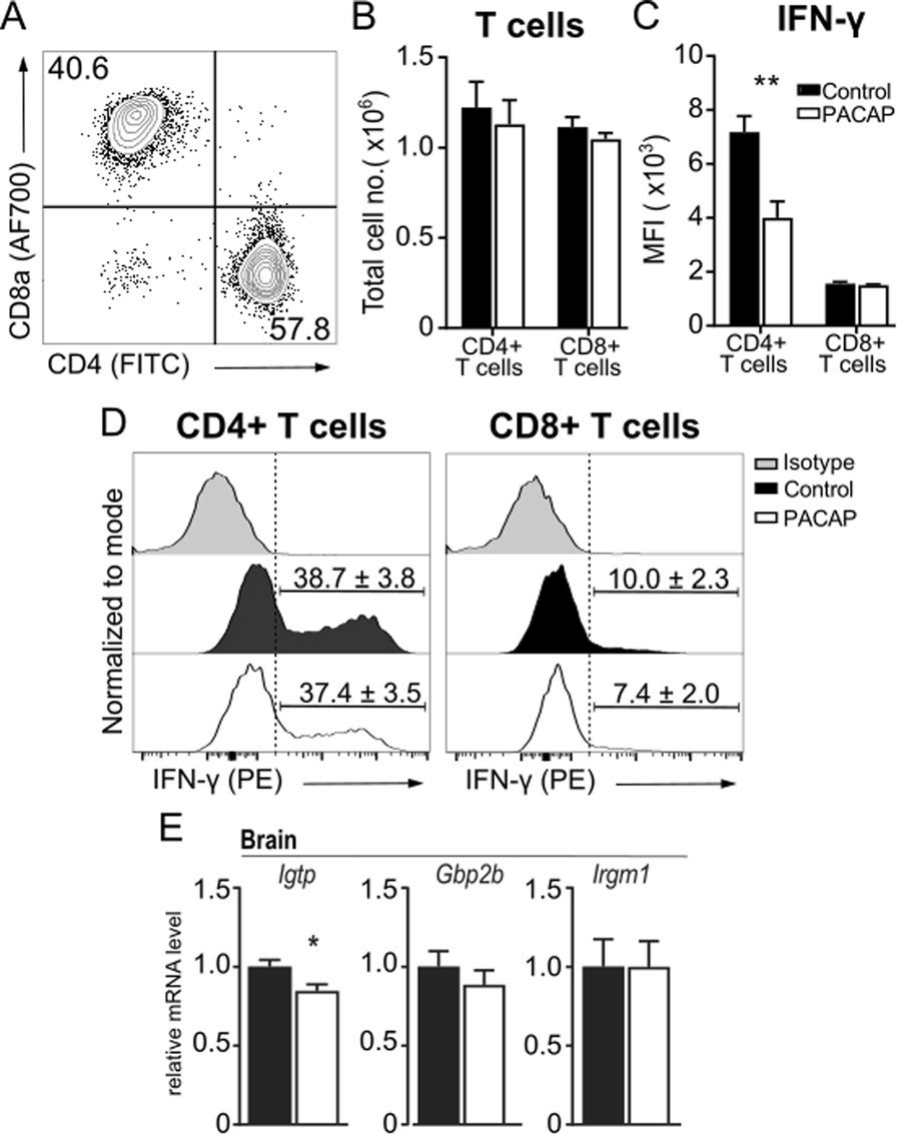
Lymphocyte infiltration into brain, IFN-γ production and host-defense factors. Immune cells were isolated from brain, and identified as single, live, CD45^hi^CD11b^−^CD3^+^ lymphocytes as shown above. **A** Representative gating illustrates discrimination of CD4^+^ and CD8^+^ T cells, and values in quadrant gates indicate cell frequency from parent population. **B** Total cell number of recruited CD4^+^ and CD8^+^ lymphocytes to the brain. **C**–**D** Intracellular analysis of IFN-γ production. Bar charts present MFI values as mean + SEM, *n* = 4, **p < 0.01 (unpaired two-tailed *t*-test). Histograms show the representative IFN-γ production by CD4^+^ and CD8^+^ T cells in comparison to isotype-control. Bars and numbers above histograms indicate mean percentage of positively stained cells + SEM. **E** Gene expression levels of host-defense factors in the whole brain homogenate were assessed using RT-*q*PCR. Bar charts represent mean values + SEM obtained in two independent experiments and analyzed together, *n* = 4–5 per experiment, *p < 0.05 (Mann–Whitney *U*-test). Control (black bars) and PACAP-treated (white bars)

Next, we analyzed the impact of PACAP on the lymphoid T cells recruited to the brain upon *T. gondii* infection. Upon chronic toxoplasmosis, T cell-derived IFN-γ production represents the major driving force to parasite control, and it is mainly mediated by CD4^+^ and CD8^+^ T cells [63–65]. Although no difference in parasite burden was previously detected in PACAP-treated brains, IFN-γ transcripts were reduced, as well as the expression of CXCL9 and CXCL10 chemokines, particularly important to T cell recruitment into the brain upon cerebral toxoplasmosis [66] (Fig. 3I). For this reason, we further investigated whether PACAP would affect T cell recruitment and their IFN-γ production. No difference was found in the total cell number of CD4^+^ and CD8^+^ T cells between the treated and control group (Fig. 4A, B). In contrast, the intracellular IFN-γ production was significantly decreased in CD4^+^ T cells upon PACAP treatment, whereas it remained unaltered in CD8^+^ T cells (Fig. 4C, D). As IFN-γ signaling regulates cell-intrinsic host defense factors against intracellular parasites, we analyzed the expression of *Igtp*, *Gbp2b* and *Irgm1* in the brain tissue, and despite the reduction of IFN-γ, only *Igtp* showed reduced expression (Fig. 4E). Thus, PACAP treatment did not alter recruitment of T cells, but reduced IFN-γ production by CD4^+^ T cells

### PACAP modulates BDNF and p75^NTR^ levels and modifies infection-induced synaptic imbalance

**Fig. 5:**
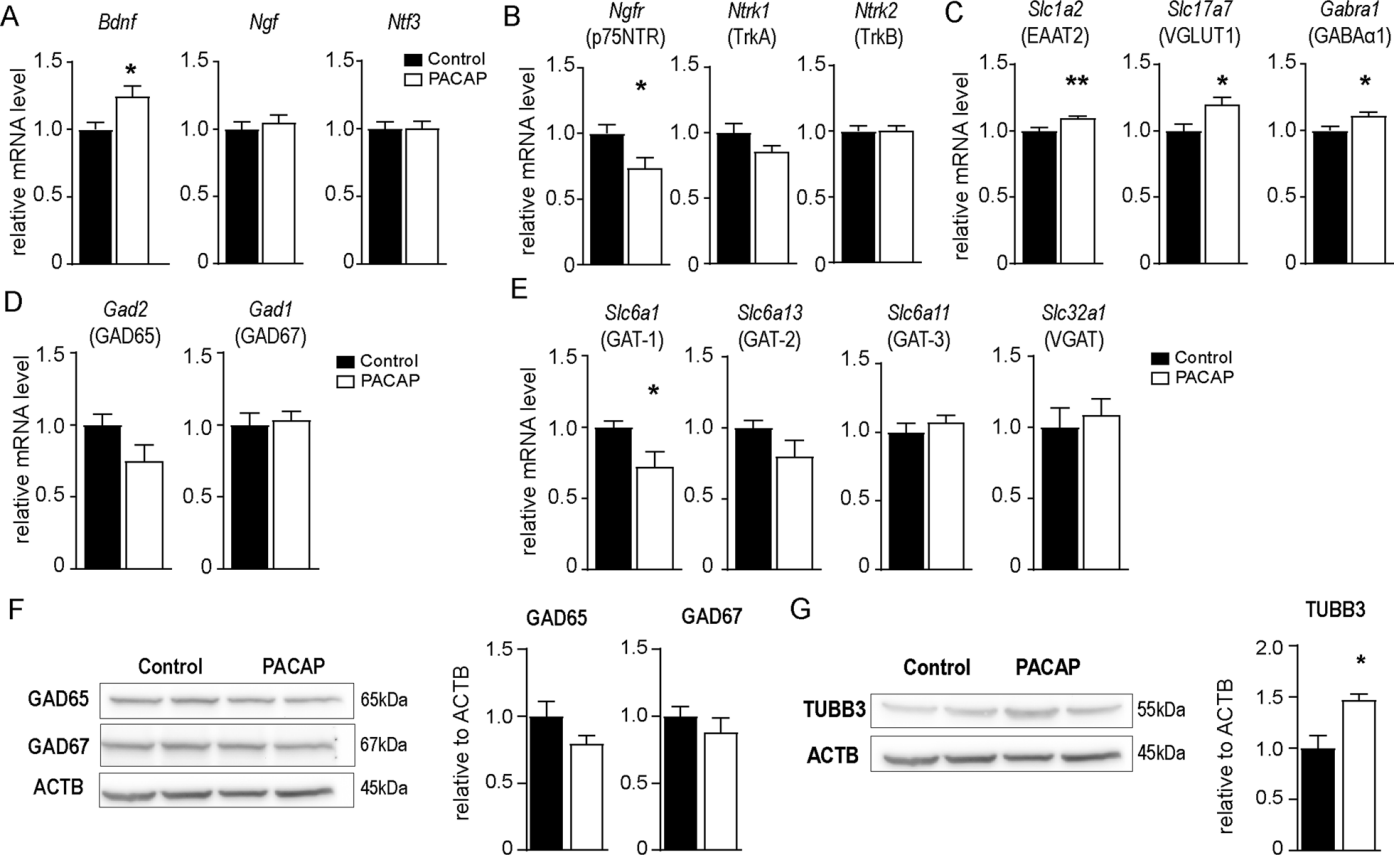
Expression levels of neurotrophins, neurotrophin-receptors and functional neuronal markers. Overall brain gene expression levels of **A** neurotrophins and **B** neurotrophin-receptors; **C** gene expression levels of glutamate transporters (EAAT2, VGLUT1) and GABAα1 subunit; **D** expression of glutamate decarboxylases GAD65 and GAD67 and **F** western blot analysis; **E** expression levels of GABA transporters (GAT-1, GAT-2, GAT-3, VGAT); **G** protein levels of beta-III-tubulin (TUBB3). Bar charts represent mean values + SEM obtained in two independent experiments and analyzed together, *n* = 4–5 per experiment, *p < 0.05, **p < 0.01 (Mann–Whitney *U*-test); Control (black bars) and PACAP-treated (white bars). Western blot membranes show two representative samples of each group, and bar charts represent mean values + SEM, *n* = 4

As the inflammatory response during cerebral toxoplasmosis is known to modify neuronal function and synapse composition [9, 14–16], we next set out to investigate whether the beneficial anti-inflammatory outcome of PACAP extended to neuronal survival and improved synaptic plasticity in the brain. In previous studies, PACAP has been associated with neurotrophins and their respective receptors [67], which are crucial for growth, development, and survival of neuronal tissue [68]. Therefore, we analyzed the transcriptional levels of the neurotrophins BDNF, NGF, NT-3, and the neurotrophin receptors p75^NTR^, TrkA, and TrkB in the brain of PACAP-treated animals (Fig. 5A-B). When compared to control group, PACAP treatment increased BDNF transcriptional levels (Fig. 5A), a crucial factor for neuronal function [69], and simultaneously reduced expression of p75^NTR^ (Fig. 5B), a receptor associated with neuronal cell death and reduced synaptic function [70, 71], but not the other neurotrophin receptors (Fig. 5B). TrkB, the main receptor for BDNF also showed no difference at protein levels (Additional file 2). Those findings prompted us to evaluate whether PACAP could compensate the reduction on synaptic alterations caused by cerebral immune response against *T. gondii*, particularly in the glutamatergic neurotransmission pathways previously described [16]. We detected increased transcriptional levels of the glutamate transporter EAAT2 and vesicular glutamate transporter VGLUT1, both implicated in neuronal protection and avoidance of excessive excitatory neuronal stimuli [72, 73] (Fig. 5C). Furthermore, we detected increased transcriptional levels of the α1 subunit of the GABA-a post-synaptic receptor (GABAAα1), an important component of the inhibitory GABAergic signaling, suggesting that PACAP probably try to counter-balance exacerbated excitatory neuronal signaling (Fig. 5C). Those results prompted us to extend our analysis to the glutamate–GABA axis. Accordingly, PACAP had no evident effect on glutamate decarboxylases GAD65 and GAD67, responsible for the most GABA amount synthesized from glutamate in the mouse brain [74] (Fig. 5D, F). Further on, we found a reduced transcriptional level of GAT-1 (Fig. 5E), a critical transporter implicate in the availability of GABA in the synaptic cleft and associated functions [75, 76]. No difference was detected for the other investigated transporters (GAT-2, GAT-3, VGAT). Of note, GAT-1 and GAT-2 were found more expressed in the brain upon *T. gondii* infection, while GAT-3 and VGAT were less expressed Additional file 1, as previous depicted for other neuronal markers upon infection [15, 16]. At last, we found increased levels of the specific neuronal component β-III tubulin (TUBB3) in the PACAP-treated brains, which in addition to transcriptional VGLUT1 levels indicates a reduced neurological damage, as previously described for chronic *T. gondii* infection [77]. Together, our results suggest that PACAP promoted neuronal viability likely via BDNF/p75^NTR^ modulation, and can modify synaptic excitatory/inhibitory neuronal imbalance present upon *T. gondii*-induced neuroinflammation.

## Discussion

Cerebral infection with *T. gondii* causes chronic inflammation leading to neuronal alterations and behavioral changes of the host. The elicited immune response is essential for parasite control in the brain, although simultaneously entails damage to the neuronal tissue. Here, we demonstrate the beneficial effects of the neuropeptide PACAP on infection-induced neuroinflammation in the murine model of cerebral toxoplasmosis. PACAP administration reduced brain pathology, microglia activation, and infiltration of monocytes, diminished production of pro-inflammatory cytokines, and bolstered neuronal protection. In previous studies, PACAP was able to modify clinical symptomatology of certain neurological disorders for example Parkinson’s [40, 41], Alzheimer’s [42], Huntington’s disease [43], and traumatic brain injury [44]. Additionally, administration of PACAP has been shown to modulate inflammation in murine models of septic shock, rheumatoid arthritis, Crohn’s disease, and multiple sclerosis [78–84]. In fact, although exerting a prominent impairment of inflammation, the immuno-modulatory ability of PACAP was found to be ambiguous, alternating from inhibitory to stimulatory effects [47]. Moreover, as modulator of microbial infections, PACAP has exhibited direct antimicrobial activity against bacteria and fungi [85]. In support, our previous studies indicate as well a reduction of *T. gondii* parasite burden in the acute phase of the infection in the periphery, through an indirect immunomodulatory anti-parasitic effect [39].

In our experimental model of cerebral toxoplasmosis, PACAP reduced the number of apoptotic caspase-3^+^ cells in the brain parenchyma. According to previous studies, the beneficial effects of PACAP on neurological diseases are mainly attributed to inhibition of caspase-3 activation mediated by receptor PAC1R [86], which is expressed by inhibitory neurons, astrocytes, and microglia [87]. Therefore, we suggest that PACAP treatment may act via PAC1R to reduce brain cell death upon *T. gondii* cerebral infection. Moreover, our histopathological examination and flow cytometric analysis indicates reduction of inflammatory foci and infiltration of immune cells in the infected brains following PACAP treatment. Likely, this immunomodulation is a secondary branch-effect of PACAP, also mediated by binding to PAC1R or other receptors in brain parenchymal cells. Previously, we and others have described the important contribution of recruited peripheral immune cells to control parasite burden in the brain in the cerebral toxoplasma model [11, 12, 88]. Surprisingly, we observed that restraining immune cell infiltration into the brain did not affect parasite control and stage conversion. Parasite levels in the periphery remained constant in the PACAP-treated group as well. In the acute toxoplasma model, we have previously detected reduced parasite burden by PACAP immunomodulation of mononuclear cells, enhancing their phagocytic capacity, although PACAP did not interfere directly with parasite replication in vitro [39]. During acute infection, the fast replicating tachyzoites are the predominant life stage of *T. gondii*, and are susceptible to immune recognition and elimination, as well as to known therapeutic drugs [25]. In contrast, during chronic infection, tachyzoites that escaped the initial immune response convert into bradyzoites, which form cysts within a variety of tissues including brain and muscles [10]. *T. gondii* cysts are shielded from therapeutic drugs and evade host immune responses [89], which suggests they are also protected from the immune response of cells modulated by PACAP treatment, and therefore could explain no difference in parasite burden. Altogether, PACAP provided neuroprotection and markedly modulated immune cell behavior without compromising parasite control.

In addition to the reduced infiltration of myeloid cells into the brain, PACAP modified the activation status and cytokine production of brain immune cells, pointing towards an anti-inflammatory effect. Markedly, Ly6C^hi^ inflammatory monocytes were reduced in number and activation status, exhibiting reduced MHCII expression. Additionally, PACAP slightly diminished levels of pro-inflammatory mediator iNOS and TNF by Ly6C^int^ monocytes-derived DCs, while iNOS and IL-1β levels were reduced by Ly6C^lo^ monocyte-derived macrophages. In our previous studies, we demonstrated the critical importance of Ly6C^hi^ monocytes upon cerebral toxoplasmosis [11]. In fact, antibody-mediated ablation of CCR2^+^ Ly6C^hi^ monocytes increased parasite burden and decreased survival of infected mice, also ablating Ly6C^hi^ monocytes in the blood [11]. In contrast, PACAP administration did not alter the presence of monocytes in the periphery, but still restricted their infiltration into the brain, reducing inflammation without compromising parasite control. Therefore, we hypothesize that PACAP exerted a specific immunomodulatory effect on the infiltrating monocytes to prevent CNS immunopathology. We have previously demonstrated that PACAP restricted the recruitment of monocytes and neutrophils, reduced their expression of pro-inflammatory cytokines, and enhanced their phagocytic capabilities upon *T. gondii* acute infection [39]. Here we additionally detected that PACAP treatment diminished CCL2 levels in the brain, which can further reduce myeloid cell infiltration. We suggest that anti-inflammatory effects of PACAP in the brain involve microglia modulation, and overall shift of the local brain inflammatory environment. In our dataset, PACAP reduced microglial MHCII expression and their production of pro-inflammatory mediators iNOS, TNF, and IL-1β. Similarly, in vitro studies have reported anti-inflammatory effects of PACAP on microglial cells, which was demonstrated by reduction of iNOS and IL-1β production in an LPS-sepsis model [90].

Further investigation on the inflammatory brain environment upon *T. gondii* infection interestingly showed that exogenous PACAP did not interfere with T cell infiltration into the brain, although it reduced IFN-γ intracellular production by CD4^+^ T cells, and the overall brain expression of CXCL9 and CXCL10. During cerebral toxoplasmosis, CD4^+^ and CD8^+^ T cells are the major source of IFN-γ in the brain, directly controlling parasite replication [91, 92]. Pointed out by previous studies, CD4^+^ T cells are targets for PACAP regulation, indicating inhibition of Th1 and favoring of Th2 differentiation and response [93–95]. Moreover, in vitro experiments indicated that macrophages and DCs treated with PACAP induce Th2 cytokines and inhibit IFN-γ in primed CD4^+^ T cells [94, 96]. Besides, our analysis showed reduction of the IFN-stimulated host-defense factor *Igtp*, even though the parasite burden was not altered. In our dataset, the absence of the modulation by PACAP on the recruitment of T cell can be explained by the late administration of the neuropeptide at the third week post-infection, when the pathology is already established in the CNS. This hypothesis is supported by previous studies, where peripheral depletion of CD4^+^ and CD8^+^ T cells did not show any effect on intracerebral T cell number, indicating that this recruitment was negligible after acute infection [65]. The reduction of IFN-γ production and *Igtp* expression suggest that PACAP may not abolish the strong Th1 response induced by *T. gondii*, but can restrain brain immunopathology.

Overall, the neuroprotective and immunomodulatory effects of PACAP are mostly attributed to the complex and wide distribution of its receptors in a variety of cell types, including neurons and immune cells. Upon inflammation, most of inhibitory immuno-modulation of PACAP targets pro-inflammatory signaling cascades including NF-κB and MAPK pathways, consequently downregulating an array of cytokines and chemokines produced by innate immune cells [47]. Our previous data have shown that PACAP administration increased expression levels of its own receptors VPAC1R and VPAC2R in immune cells, which were followed by reduced infiltration of monocytes and neutrophils, and reduced pro-inflammatory cytokine expression during acute toxoplasmosis [39]. Additionally, several studies indicate that the anti-inflammatory activity of PACAP on macrophages, monocytes, and DCs are primarily exerted through VPAC1R [97–100]. While VPAC1R is constitutively expressed in those cells, VPAC2R is induced in lymphocytes, monocytes, and macrophages after inflammatory stimulation, and has been linked to increased Th2 response [93–95]. Aligned with our finding that PACAP reduced IFN-γ production by CD4^+^ T cells in the brain, studies with transgenic mice overexpressing the receptor VPAC2R in CD4^+^ T cells developed increased Th2 responses, while the Th1 response prevailed in VPAC2R-deficient mice [95, 101]. Of note, VPAC1R and VPAC2R were also found expressed by the newly discovered innate lymphoid cells 2 (ILC2), the innate-correlated cells of the Th2 adaptive immune response, showing to be important for immune response [102]. In microglia, PAC1R is the main receptor mediating anti-inflammatory effects of PACAP, inhibiting NO production during ischemia, reducing pro-inflammatory cytokine release, and inducing a microglial phenotype transformation associated to regenerative growth, tissue repair and clearance of local cellular debris [45, 103]. Taken together, we suggest that the beneficial effects of PACAP upon cerebral toxoplasmosis rely partially on the differential expression of PACAP receptors on the immune cells directly involved in the disease control.

Further on, we demonstrate that PACAP exerted not only beneficial anti-inflammatory effects on cerebral toxoplasmosis, but also showed a neuroprotective outcome in the infected brain parenchyma. Interestingly, we found higher transcriptional levels of the neurotrophin BDNF in the brain of PACAP-treated mice, and no differences were detected for NGF or NT-3. In fact, PACAP-mediated neuroprotection have been attributed to enhanced expression of neurotrophins and related receptors via PAC1R signaling [33]. Of note, PAC1R is considered the main PACAP receptor in the brain, and the one with the highest affinity for PACAP [104]. In terms of neurotrophins, BDNF is crucial for neuronal survival, and its expression was also found reduced upon neurodegeneration [105] and cerebral toxoplasmosis [16]. Indeed, in vitro studies on primary neuronal cultures revealed that PACAP stimulates and recovers BDNF expression upon injury conditions, mainly via PAC1R [67, 106]. Additionally, reduced hippocampal BDNF was also detected in PAC1R knockout mice [107]. Overall, enhancement of BDNF expression is proposed as a key mechanism in neuroprotection and rescue of cognitive impairment via PAC1R [33]. Since PACAP affected BDNF transcriptional levels, we sought to further investigate the modulation of neurotrophin-related receptors. BDNF mainly binds to TrkB (*Ntrk2*), NGF to TrkA (*Ntrk1*), and the binding of neurotrophins to p75^NTR^ (*Ngfr*) is more promiscuous and complex, and it is thought to define the affinity of the neurotrophins and their precursors to other receptors [68]. In fact, p75^NTR^ has been nominated as death-receptor, and binding of the BDNF precursor to p75^NTR^ has shown to induce cell death and reduce synaptic function [108]. Surprisingly, we found that PACAP reduced expression of p75^NTR^ in the brain, and did not alter TrkB levels, suggesting an additional contribution of PACAP to neuronal survival via BDNF–p75^NTR^ axis modulation. Other studies in stroke models have shown that PACAP reduced p75^NTR^ levels, regulated the activity of TrkB by increased phosphorylation, and consequently increased affinity for BDNF [109, 110]. We have previously demonstrated that p75^NTR^ signaling not only shaped brain neuronal architecture, but also altered the behavior of innate immune cells during neuroinflammation [52]. Moreover, we have previously found a beneficial association of p75^NTR^ and immune cells upon PACAP treatment of acute *T. gondii* infection, in which reduced expression of p75^NTR^ on Ly6C^hi^ inflammatory monocytes and increased overall BDNF gene expression was associated with reduction on pro-inflammatory mediators and enhanced parasite elimination [39]. Therefore, we suggest that PACAP-mediated neuroprotective effects and immunomodulatory properties provide a beneficial overlap via modulation of BDNF/p75^NTR^ axis to counteract *T. gondii* neuroinflammation. In addition, PACAP increased the levels of TUBB3, a specific cytoskeleton component of neurons shown to be modified upon cerebral *T. gondii* infection [77], reinforcing PACAP contribution to neuronal survival. Still, future studies are necessary to address the involvement of PACAP, BDNF precursors and p75^NTR^ function in immune cells within the brain.

Besides neurotrophins, PACAP-treated mice showed an increase in the expression levels of synaptic markers EAAT2 and VGLUT1 from neuronal glutamatergic pathway, and GABAAα1 from inhibitory GABAergic pathway. As recently shown in our previous studies, the expression of these synaptic markers was significantly decreased upon cerebral toxoplasmosis, suggesting that *T. gondii* neuroinflammation shifted the synaptic excitation–inhibition balance towards excitotoxicity at the transcriptional [16] and protein level [15]. Detrimental alterations in the glutamatergic and GABAergic signaling upon *T. gondii* infection have also been further characterized, and suggest a neurodegenerative state of the infected CNS [77, 111]. Here, PACAP’s overall reduction of brain inflammation without dysregulation of parasite control points towards a less dysfunctional neuronal network. The explanation relies on the reduced IFN-γ production, MHCII expression, NO production and other pro-inflammatory cytokines in the brain, which have detrimental effects on neurons [112]. Antibody-mediated depletion of IFN-γ during cerebral toxoplasmosis partially recovered synaptic alterations, although it triggered an uncontrolled parasite burden in the brain [16]. Therefore, PACAP exerts amelioration of brain immunopathology and consequently reduces synaptic dysfunction.

Finally, it is important to point out the limitations of our study. Exogenous administration of PACAP is sought to recover and/or enhance the beneficial effects of the endogenous presence of the neuropeptide, which are diminished or lost during neuroinflammation [45]. We applied PACAP via i.p. injections, and despite the relatively short half-life in the organism, we achieved neuroprotective results. In recent studies, intranasal administration of PACAP has proven to deliver more efficiently the neuropeptide to the brain, showing rapid absorption via nasal mucosa and high uptake in the occipital cortex and striatum [113, 114]. Still, PACAP is transported directly across the BBB in a very rapid transport rate [50, 115], and its uptake was found particularly high in the hypothalamus and hippocampus [116]. Besides the administration route and brain-region-specific penetration, another aspect not explored in this study is the effect of PACAP specific on astrocytes, which are known to contribute to recover the neuronal homeostasis during cerebral toxoplasmosis. We believe that PACAP restriction of myeloid immune cell infiltration and the shifted brain inflammatory environment, together, can be partially mediated by the effect of PACAP on astrocytes, once they also express PACAP receptors. Moreover, a comprehensive characterization of the PACAP receptors and neurotrophin receptors on Ly6C^+^ monocytes and on the recently described ILCs are necessary for the understanding of the neuro-immune mechanism involved in neuroinflammation and neurodegeneration.

## Conclusions

In summary, exogenous administration of PACAP ameliorated *T. gondii* infection-induced brain pathology, restrained the recruitment of peripheral myeloid cells to the brain, and reduced the activation status and production of pro-inflammatory mediators by microglia and monocytes, resulting in neuroprotection. This immunomodulation restricted the detrimental effects of neuroinflammation. Moreover, PACAP promoted neuronal health likely via BDNF/p75^NTR^ axis modulation, resulting in diminished dysregulation of the neuronal network with implications on glutamatergic and GABAergic signaling inflicted by cerebral toxoplasmosis. Together, our findings unravel that exogenous PACAP administration provides beneficial cumulative effects of neuroprotection and immunomodulation to overcome infection-induced neuroinflammation.

## Supplementary Information

**Additional file 1.** Complementary transcriptional levels of PACAP receptors and neuronal markers. Overall brain gene expression levels for (A) PACAP receptors, (B) glutamate decarboxylases GAD65 and GAD67, and (C) GABA receptors. Additional naïve dataset was introduced as comparison to previously unknown transcriptional levels in the brain of the analyzed genes, but the values were normalized by the control group (infected) as shown in the previous figures for appropriate comparison. Bar charts represent mean values + SEM obtained in two independent experiments and were analyzed together, n = 4-5 per experiment, *p < 0.05, **p < 0.01, ***p < 0.001, ****p < 0.0001 (ANOVA with Tukey correction); Naïve (gray bars), control (black bars) and PACAP-treated (white bars).

**Additional file 2.** Complementary protein levels of neurotrophin receptor TrkB. Overall brain expression levels of TrkB in control vs PACAP-treated animals. Western blot membrane shows two representative samples of each group, and bar charts represent mean values + SEM, n = 4.

## Data Availability

The datasets used and/or analyzed during the current study are available from the corresponding author on reasonable request.

## Abbreviations

AC: Adenylate cyclase
AKT: Protein kinase B
ANOVA: Analysis of variance
BBB: Blood–brain barrier
BDNF: Brain-derived neurotrophic factor
cAMP: Cyclic adenosine monophosphate
Casp3: Cleaved caspase-3
CCL2: Chemokine (C–C motif) ligand 2
CD: Cluster of differentiation
CNS: Central nervous system
DCs: Dendritic cells
DNA: Deoxyribonucleic acid
EAAT2: Excitatory amino acid transporter 2
FACS: Fluorescence-activated cell sorting
FBS: Fetal bovine serum
FMO: Fluorescence minus one
GABA: Gamma-aminobutyric acid
Gbp2b: Guanylate binding protein 1
GPCRs: G protein-coupled receptors
H&E: Hematoxylin and eosin
HBSS: Hanks' balanced salt solution
HEPES: 4-(2-Hydroxyethyl)-1-piperazineethanesulfonic acid
IFN-γ: Interferon gamma
Igtp: Interferon gamma-induced GTPase
IL-6: Interleukin 6
iNOS: Inducible nitric oxide synthase
IP3: Phosphatidylinositol 3 kinase
Irgm1: Immunity-related GTPase family M protein 1
MHCII: Major histocompatibility complex type II
LPS: Lipopolysaccharides
NGF: Nerve growth factor
NO: Nitric oxide
NT-3: Neurotrophin-3
p75NTR: P75 neurotrophin receptor
PACAP: Pituitary Adenylate Cyclase-Activating Polypeptide
PBS: Phosphate-buffered saline
PCR: Polymerase chain reaction
PFA: Paraformaldehyde
PKA: Protein kinase A
PKC: Protein kinase C
PLC: Phospholipase C
RNA: Ribonucleic acid
RT-qPCR: Quantitative reverse transcription PCR
SEM: Standard error of the mean
TLA: Toxoplasma lysate antigen
TNF: Tumor necrosis factor
TrkA: Tropomyosin receptor kinase A
TrkB: Tropomyosin receptor kinase B
VGLUT1: Vesicular glutamate transporter 1
