# Supplementary figures and images for: The neuropeptide PACAP alleviates T. gondii infection-induced neuroinflammation and neuronal impairment
Source: J Neuroinflammation. 2022 Nov 19;19:274. doi: 10.1186/s12974-022-02639-z (PMC9675261; doi:10.1186/s12974-022-02639-z)

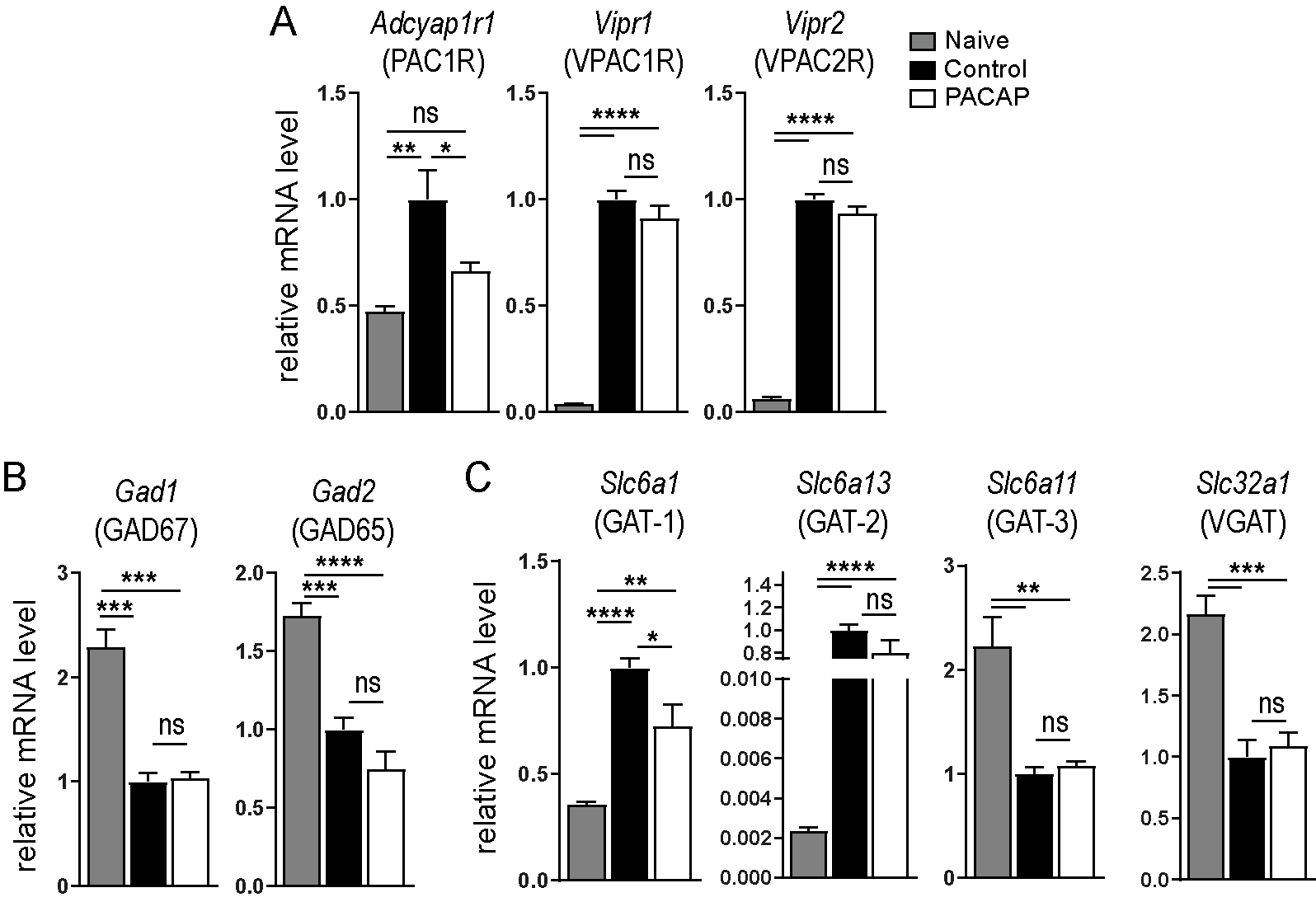

Supplement: Supplementary file 1 — Additional file 1. Complementary transcriptional levels of PACAP receptors and neuronal markers. Overall brain gene expression levels for (A) PACAP receptors, (B) glutamate decarboxylases GAD65 and GAD67, and (C) GABA receptors. Additional naïve dataset was introduced as comparison to previously unknown transcriptional levels in the brain of the analyzed genes, but the values were normalized by the control group (infected) as shown in the previous figures for appropriate comparison. Bar charts represent mean values + SEM obtained in two independent experiments and were analyzed together, n = 4-5 per experiment, *p < 0.05, **p < 0.01, ***p < 0.001, ****p < 0.0001 (ANOVA with Tukey correction); Naïve (gray bars), control (black bars) and PACAP-treated (white bars). [file 12974_2022_2639_MOESM1_ESM.tif]

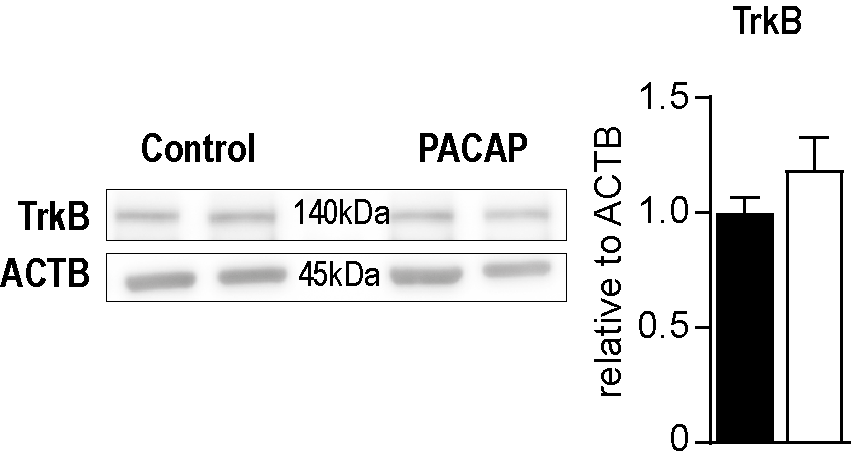

Supplement: Supplementary file 2 — Additional file 2. Complementary protein levels of neurotrophin receptor TrkB. Overall brain expression levels of TrkB in control vs PACAP-treated animals. Western blot membrane shows two representative samples of each group, and bar charts represent mean values + SEM, n = 4. [file 12974_2022_2639_MOESM2_ESM.tif]
